# The relationship between scoliosis and balance in a population of adolescents with AIS

**DOI:** 10.1186/1748-7161-8-S1-O5

**Published:** 2013-06-03

**Authors:** P Knott, J Musto, S Thompson, S Mardjetko

**Affiliations:** 1 Rosalind Franklin University, Chicago, USA; 2 Illinois Bone and Joint Institute, Morton Grove, USA

## Background

The etiology of Adolescent Idiopathic Scoliosis has been investigated for years. Now that the genetic basis for the disease has been established, the actual mechanism for production of the curve still needs to be discovered. Several have proposed a theory involving balance, with the brain and spinal cord causing spinal curvature related to neurologic signals [1,2]. An opportunity to observe spinal curvature and balance simultaneously exists when using the Formetric 4D (Diers Medical Systems) to assess spinal deformity. As surface topography measurements are being taken on the standing patient, force plate data on balance is also being generated. This study looked at the balance data in patients being measured for scoliosis, to see if there was a correlation between balance and spinal deformity measurements.

## Goals

To look for a relationship between balance data and spinal deformity measurements in adolescent patients with idiopathic scoliosis.

## Methods

One hundred thirty patients were measured a total of 188 times using the Formetric 4D to evaluate their spinal deformity. While doing this, a 6-second balance test was also performed using the Pedoscan force plate integrated with the Formetric. The deformity measurements considered were Thoracic and Lumbar Scoliosis Angles, Thoracic Kyphosis, Lumbar Lordosis, Sagittal Vertical Axis, and Coronal Vertical Axis. The balance measurements considered were Center of Pressure (COP) Total Excursion, Mean Velocity of COP Movement, A/P COP Movement, and Lateral COP Movement. Correlation coefficients were calculated for each pair of deformity and balance measurements.

## Results

There were no significant correlations between any of the balance measurements and the magnitude of the deformity measurements in this population.

## Conclusions

This study was not able to demonstrate that patients with larger deformity measurements also had more difficulty maintaining their balance. A number of future research possibilities are suggested to further evaluate this potential relationship.
